# Points in Prescribing

**Published:** 1908-09-26

**Authors:** D. M. Macdonald

**Affiliations:** Dunkeld.


### THE GENERAL PRACTITIONER'S COLUMN.

**[Contributions to this Column are invited, and if accepted will be paid for.]**

### POINTS IN PRESCRIBING.

By D. M. MACDONALD, M.D., Dunkeld.

In this paper the reader's attention is drawn to some common and not unimportant details to aid the presentation of certain remedies in an appropriate and palatable form.

Take, for example, the cachet or rice-paper wafer. Many of the difficultly diffusible powders given in mixture form, and for the most part insoluble, may be given in cachet form. Such drugs are salol, salicylate of quinine, rhubarb, calomel, and bismuth salts, which frequently get into the crevices and bottom of the bottle and are with difficulty dislodged.

The patient should be told to dip the cachet between the finger and thumb into water till it becomes pulpy, before placing it on the tongue. If the cachet is placed on the tongue without being previously moistened, with some patients who are nervous about swallowing medicine, the results are sometimes embarrassing. There can be no doubt that in many ways the cachet is a distinctly better form of administration than the tablet or the pill, because it is more rapidly dissolved.

Perhaps one of the greatest difficulties of the practitioner is to write a formula for an emulsion. In many instances the substance to be emulsified is given, and its combination left to the discretion of the dispenser. In some ways this is an advantage, but it is open to the objection that, if the patient travels, the consistence and taste may vary extremely. Castor oil may frequently be suitably given in this form. Take the following formula for example:—

| | | |
|---|---|---|
| ℞ | Ol. Ricini ... ... ... ... ... ... | ʒiv. |
| | Pulv. Acaciæ ... ... ... ... ... | gr. 80 |
| | Ol. Caryophylli ... ... ... ... ... | ♏ij. |
| | Aq. Menth. Pip. ad ... ... ... ... | ℥iss. |
| M. Ft. Haustus. | | |

By placing the oil in a mortar, adding the acacia and three drachms of water all at once, then stirring, and after a little adding the rest of the water, a pleasant vehicle for castor oil results, which may be taken in ignorance even by those to whom the oil is most repugnant. For the summer diarrhœa of children it is most useful, given in divided doses, and in tubercular diarrhœa creosote or perchloride of mercury may be added.

A simple and convenient method of administering calomel to children who object to medicine is to give it in tablet form combined with sugar. If these are kept, not in a box, but in a paper bag, children will take them without suspicion. For throat medication, pharmaceutical houses turn out legions of combinations, but the prescriber may at any time give his own particular combination, and ask the pharmacist to use as a basis the well-known gelatine one used for glycecols, or the same basis combined with tolu and liquorice manufactured by well-known chemists. The *B.P.C.*, recently published, supplies abundance of reliable formulæ in this as in many other departments of pharmacy, and an intimate knowledge of it will help the practitioner in almost every prescription he writes, as well as suggest improvement even in the favourite combination of many prescribers. It will also help him to avoid pitfalls, such as combining aspirin with sodium bicarbonate; pepsine in combination with alkali is a very common prescription which has recently been shown by Mr. I. Tocher to be practically inert—a point worth knowing. In a short time malt extract and its different forms will be much in use. Malt extract, especially for children, may be given with meals. They will take it spread on bread instead of jam quite readily, and in this way the objection to regular administration may be almost unconsciously overcome.

It has often been pointed out that effervescent preparations act quickly on the body. A combination of antipyrin, potassium bromide, and caffeine citrate given in effervescent form is a ready specific for many forms of headache, neuralgia, etc. Pharmaceutical chemists and others now prepare effervescent powders from prescriptions, and in this form they are palatable and efficacious and readily taken by patients.

